# Health literacy domains and socioeconomic inequalities in leisure-time physical activity among Korean adults

**DOI:** 10.3389/fpubh.2026.1846028

**Published:** 2026-05-28

**Authors:** Myong-Won Seo

**Affiliations:** Departments of Sports and Leisure Studies, College of Physical Education, Keimyung University, Daegu, Republic of Korea

**Keywords:** domain-specific health literacy, educational inequality, guideline adherence, health promotion, income disparity

## Abstract

**Purpose:**

Health literacy is an important determinant of health behavior and may contribute to socioeconomic inequalities in health-promoting behaviors. However, evidence remains limited on how total and domain-specific health literacy relate to leisure-time physical activity (LTPA) adherence and whether socioeconomic gradients in health literacy are reflected in disparities in LTPA among Korean adults.

**Methods:**

This cross-sectional study used data from 3,551 Korean adults aged 18–64 years who participated in the 2024 Korea National Health and Nutrition Examination Survey. LTPA was assessed using the Korean version of the Global Physical Activity Questionnaire and categorized as adherent or non-adherent according to the threshold of 150 min/week. Health literacy was assessed using a 10-item multidimensional instrument covering disease prevention, health promotion, healthcare, and technology and resources. Multivariable logistic regression analyses were conducted to estimate odds ratios (ORs) and 95% confidence interval (95% CIs) for LTPA adherence according to total and domain-specific health literacy. Socioeconomic differences in health literacy and trends in LTPA adherence across health literacy quartiles were also examined.

**Results:**

Of the 3,551 participants, 24.6% met the LTPA guideline. Higher total health literacy was independently associated with higher odds of meeting the LTPA guideline across all models (Model 1: OR, 1.066; Model 2: OR, 1.058; Model 3: OR, 1.056; all *p* < 0.001). All four health literacy domains were positively associated with LTPA adherence, with the strongest association observed for the health promotion domain (Model 3: OR, 1.585; *p* < 0.001). Health literacy showed clear gradients across household income and education levels, and LTPA adherence increased progressively across health literacy quartiles (*P* for trend < 0.001).

**Conclusion:**

Higher health literacy was independently associated with greater adherence to LTPA guidelines among Korean adults and showed a clear socioeconomic gradient. Health literacy may represent both a behavioral determinant of LTPA and a pathway linking socioeconomic disadvantage to disparities in health-promoting activity.

## Introduction

Regular physical activity is a key determinant of overall health and is recommended as a strategy for the prevention of noncommunicable diseases (1). World Health Organization (WHO) guidelines recommend that adults engage in at least 150–300 min of moderate-intensity aerobic physical activity, 75–150 min of vigorous-intensity activity, or an equivalent combination each week (1). However, insufficient physical activity remains common, and understanding the factors that shape adherence to recommended activity levels remains an important public health priority (2).

Among the domains of physical activity, leisure-time physical activity (LTPA) is important because it is performed outside occupational demands and is associated with health outcomes distinct from those of occupational physical activity (3, 4). Holtermann et al. (5) reported that occupational physical activity and leisure-time physical activity were associated with different cardiovascular health outcomes. In addition, Seo et al. (3) demonstrated a dose–response association between LTPA and metabolic syndrome risk, whereas occupational physical activity showed no significant non-linear association. Taken together, these findings support the clinical relevance of LTPA and highlight the importance of identifying modifiable determinants of LTPA adherence. In the present study, LTPA was selected as the primary outcome because it reflects discretionary physical activity undertaken during non-working time and may therefore be more sensitive to differences in health literacy than occupational physical activity.

Health literacy is a key determinant of health behavior and refers to the ability to access, understand, appraise, and use health information for health-related decision making (6, 7). Its relevance extends beyond knowledge acquisition to competencies required for disease prevention, health promotion, navigation of healthcare systems, and interpretation of health information from digital and media sources (7, 8). These competencies are particularly relevant to LTPA, which relies on individual agency and sustained behavioral engagement. Consistent with this evidence, a systematic review found that higher health literacy was generally associated with higher levels of physical activity (9).

Although previous studies have linked overall health literacy to physical activity, little is known about whether specific domains of health literacy are differentially associated with leisure-time physical activity. Because health literacy is multidimensional, examining domain-specific associations may provide more actionable insight than evaluating a single overall score. Health literacy is socially patterned and unequally distributed across socioeconomic groups (7, 10). Lower health literacy is consistently associated with socioeconomic disadvantage, including lower income, lower educational attainment, and social vulnerability (11, 12). Such disparities may contribute to socioeconomic inequalities in health-promoting behaviors (13). However, evidence remains limited regarding the extent to which health literacy domains and socioeconomic position are associated with LTPA adherence. In particular, it is unclear whether socioeconomic gradients in health literacy correspond to disparities in adherence to LTPA. Therefore, we examined whether total and domain-specific health literacy were associated with LTPA adherence, whether health literacy scores differed according to household income and education level, and whether adherence to LTPA guidelines increased across health literacy quartiles. We hypothesized that higher health literacy would be associated with higher odds of meeting LTPA guidelines and that socioeconomic gradients in health literacy would parallel disparities in LTPA adherence.

## Methods

### Data source

This cross-sectional study used data from the 2024 Korean National Health and Nutrition Examination Survey (KNHANES), a nationally representative survey of the Korean population. Conducted annually by the Korea Disease Control and Prevention Agency (KDCA), KNHANES provides extensive data on demographic characteristics, health status, health-related behaviors, and nutritional factors. The study protocol was approved by the Institutional Review Board of the KDCA (Approval No. 2022-11-16-R-03). All study procedures were carried out in accordance with the Declaration of Helsinki. Written informed consent was obtained from all participants before the survey and blood collection.

### Participants

The present study used data from the 2024 KNHANES, obtained through the official KNHANES website. Of the 6,997 individuals who participated in the survey, those younger than 18 years and those aged 65 years or older were excluded to restrict the analysis to Korean adults. In addition, participants with missing and refusing data for any variables were excluded. After these exclusions, the final analytic sample consisted of 3,551 Korean adults. Because this study was based on secondary analysis of a nationally representative dataset, no *a priori* sample size calculation was performed. The final sample size was determined by the number of eligible participants in the 2024 KNHANES after applying the predefined inclusion and exclusion criteria. LTPA was assessed using the Korean version of the Global Physical Activity Questionnaire (K-GPAQ) (14). Weekly physical activity volume was calculated as the sum of twice the minutes of vigorous-intensity activity and the minutes of moderate-intensity activity (3). Participants were then categorized as LTPA-adherent or non-adherent based on whether they achieved at least 150 min/week of leisure-time physical activity (15).

### Demographic and health-related variables

Demographic and socioeconomic characteristics included age, sex, education level, household income, and occupation. Age was analyzed as a continuous variable and sex was categorized as male or female. Education level was classified into four groups: elementary school, middle school, high school, and undergraduate or higher. Household income was categorized into quartiles (Q1–Q4). Occupation was classified into seven categories: administrators, managers, and professionals; office workers; service workers and shop sales workers; skilled agricultural and fishery workers; machine operators and assemblers; laborers; and jobless (e.g., housewives and students). Smoking status was categorized as never smoker, former smoker, or smoker. Metabolic syndrome was defined using the updated modified National Cholesterol Education Program Adult Treatment Panel III criteria (16). To account for ethnic-specific differences in abdominal obesity, the waist circumference cut-off points recommended by the Korean Society for the Study of Obesity were applied (17). Metabolic syndrome was considered present when at least three of the following five components were identified: (1) waist circumference >90 cm in male or >85 cm in female, (2) fasting plasma glucose ≥100 mg/dL, (3) triglycerides ≥150 mg/dL, (4) high-density lipoprotein cholesterol <40 mg/dL in male or <50 mg/dL in female, and (5) systolic blood pressure ≥130 mmHg and/or diastolic blood pressure ≥85 mmHg. Sitting time represented the usual time spent sitting or reclining and was converted to minutes per week. Additionally, walking time represented the total weekly duration of walking accumulated in bouts of at least 10 min and was expressed in minutes per week.

### Health literacy

Health literacy was assessed using the 10-item health literacy instrument included in KNHANES (18). This instrument was officially developed through a KDCA policy research project in 2022 and was designed to assess health literacy across four domains: disease prevention (3 items), health promotion (1 item), healthcare (4 items), and technology and resources (2 items) (8, 18). Each item was rated on a 4-point Likert scale ranging from 1 (“never”) to 4 (“always”), and total scores were calculated by summing all item responses, yielding a possible range of 10 to 40. The instrument was previously developed and validated for use in KNHANES, with evidence supporting its multidimensional structure and validity. Detailed item descriptions are provided in Supplementary Table S1. The English version was based on the translation reported by Yoon et al. (8).

### Statistical analysis

All data are presented as mean ± standard error of the mean (SEM) for continuous variables and as number (%) for categorical variables. Differences in participant characteristics according to LTPA guideline adherence were assessed using the chi-square test for categorical variables. For continuous variables, unadjusted differences were examined using the independent *t*-test, and adjusted differences were evaluated using analysis of covariance (ANCOVA) with age and sex as covariates. Adjusted means were estimated accordingly. To examine socioeconomic differences in health literacy, mean health literacy scores were compared across household income quartiles and education levels using one-way analysis of variance (ANOVA), followed by Bonferroni-corrected *post hoc* tests. The proportion of participants meeting the LTPA guideline across health literacy quartiles was compared using the chi-square test, and a test for linear trend was conducted using multivariable logistic regression after adjustment for covariates.

Multivariable logistic regression analyses were performed to estimate odds ratios (ORs) and 95% confidence intervals (CIs) for LTPA guideline adherence according to total and domain-specific health literacy scores. Health literacy was analyzed as both a continuous variable and a categorical variable based on quartiles. Model 1 was adjusted for age and sex. Model 2 was further adjusted for education level and household income. Model 3 was additionally adjusted for smoking status and metabolic syndrome. Multicollinearity among predictors was assessed using variance inflation factors (VIFs), and model fit was evaluated using the Hosmer–Lemeshow goodness-of-fit test. All analyses were performed using R version 4.5.3 (R Foundation for Statistical Computing, Vienna, Austria), and statistical significance was set at *p* < 0.05.

## Results

The full analytic sample included 3,551 Korean adults aged 18–64 years, of whom 24.6% met the LTPA guideline. The overall demographic and health-related characteristics of the study population are presented in Table 1. Those who met the guideline were younger than non-adherent participants (*p* < 0.001), and the proportion of men was higher in the adherent group (*p* < 0.001). In addition, adherence to the LTPA guideline was associated with higher education level and household income (both *p* < 0.001). Participants in the LTPA-adherent group had a favorable overall health profile. Compared with the non-adherent group, the LTPA-adherent group showed lower prevalence of metabolic syndrome, lower sitting time, and greater walking time in both unadjusted and adjusted analyses (all *p* < 0.001). In addition, total health literacy scores were significantly higher in the LTPA-adherent group, and this difference remained significant after adjustment for age and sex (*p* < 0.001).

**Table 1:** Demographic characteristics for participants according to LTPA guideline adherence.

Variable		Total (*N* = 3,551)	LTPA non-adherent (*n* = 2,679)	LTPA-adherent (*n* = 872)	*p* value
Age	Age, M ± SEM	44.95 ± 0.22	46.31 ± 0.25	40.75 ± 0.45	<0.001
Sex	Male, *n* (%)	1,540 (43.4)	1,095 (40.9)	445 (51.0)	<0.001
	Female, *n* (%)	2011 (56.6)	1,584 (59.1)	427 (49.0)	
Education	≤Elementary School, *n* (%)	110 (3.1)	101 (3.8)	9 (1.0)	<0.001
	≤Middle School, *n* (%)	205 (5.8)	185 (6.9)	20 (2.3)	
	≤High School, *n* (%)	1,336 (37.3)	1,030 (38.4)	306 (35.1)	
	≥Undergraduate, *n* (%)	1900 (53.5)	1,363 (50.9)	537 (61.6)	
Smoking	Never smoker, *n* (%)	2,973 (83.7)	2,233 (83.4)	740 (84.9)	<0.001
	Former smoker, *n* (%)	94 (2.6)	64 (2.4)	30 (3.4)	
	Smoker, *n* (%)	484 (13.6)	382 (14.2)	102 (11.7)	
House hold income level	Quartile 1, *n* (%)	294 (8.3)	234 (8.7)	60 (6.9)	<0.001
	Quartile 2, *n* (%)	743 (20.9)	599 (22.4)	144 (16.5)	
	Quartile 3, *n* (%)	1,119 (31.5)	855 (31.9)	264 (30.3)	
	Quartile 4, *n* (%)	1,395 (39.3)	991 (31.9)	404 (46.3)	
Occupation	Administrators, managers, and professionals, *n* (%)	757 (21.3)	523 (19.5)	234 (26.8)	<0.001
	Office workers, *n* (%)	510 (14.4)	378 (14.1)	132 (15.1)	
	Service workers and shop sales workers, *n* (%)	363 (17.9)	498 (18.6)	138 (15.8)	
	Skilled agricultural and fishery workers, *n* (%)	60 (1.7)	60 (2.2)	0 (0)	
	Machine operators and assemblers, *n* (%)	403 (11.3)	318 (11.9)	85 (9.7)	
	Laborers (Not elsewhere classified), *n* (%)	222 (6.3)	192 (7.2)	30 (3.4)	
	Jobless (e.g., housewife, students), *n* (%)	963 (2)	710 (26.5)	253 (29.0)	
Metabolic syndrome, *n* (%)		714 (20.1)	592 (22.1)	122 (14.0)	<0.001
Sitting time, min/week	Unadjusted, M ± SEM	563.66 ± 5.38	569.63 ± 6.85	545.30 ± 6.07	<0.001
	Adjusted, M ± SEM		573.81 ± 6.19	532.45 ± 10.96	<0.001
Walking time, min/week	Unadjusted, M ± SEM	238.47 ± 4.27	212.52 ± 4.62	318.18 ± 9.59	<0.001
	Adjusted, M ± SEM		209.76 ± 4.84	326.66 ± 8.58	<0.001
Health literacy, score	Unadjusted, M ± SEM	31.36 ± 0.08	31.01 ± 0.09	32.43 ± 0.15	<0.001
	Adjusted, M ± SEM		31.05 ± 0.09	32.31 ± 0.16	<0.001

Values are presented as mean ± SEM (standard error of mean) for continuous variables and *n* (%) for categorical variables, LTPA, leisure-time physical activity, LTPA met indicates meeting leisure-time physical activity guidelines, Adjusted for age and sex.

In logistic regression analyses (Table 2), higher total health literacy was associated with higher odds of meeting the LTPA guideline across all models. In Model 1, each one-point increase in total health literacy score was associated with a 6.6% increase in the odds of LTPA guideline adherence after adjustment for age and sex (*p* < 0.001). The association remained significant after further adjustment for education and household income level in Model 2 (OR, 1.058; *p* < 0.001) and for smoking and metabolic syndrome in Model 3 (OR, 1.056; *p* < 0.001). Domain-specific analyses indicated significant positive associations between all four health literacy domains and LTPA adherence, with the strongest association observed for the health promotion domain (Model 3: OR, 1.585; *p* < 0.001). Multicollinearity diagnostics indicated no evidence of problematic multicollinearity among the predictors, with all adjusted VIF values below 1.3. The Hosmer–Lemeshow goodness-of-fit test indicated acceptable model fit (*p* = 0.863).

**Table 2:** Associations of total and domain-specific health literacy with LTPA guideline adherence.

Variable	Model 1			Model 2			Model 3		
	*β*	SEM	Odds ratio (95% CI)	*β*	SEM	Odds ratio (95% CI)	*β*	SEM	Odds ratio (95% CI)
Total health literacy	0.064	0.009	1.066 (1.047, 1.085) ***	0.057	0.009	1.058 (1.039, 1.078) ***	0.055	0.009	1.056 (1.037, 1.076) ***
Disease prevention	0.143	0.025	1.154 (1.099, 1.211) ***	0.125	0.025	1.133 (1.078, 1.19) ***	0.119	0.025	1.126 (1.072, 1.184) ***
Health promotion	0.516	0.071	1.675 (1.457, 1.925) ***	0.473	0.072	1.605 (1.393, 1.848) ***	0.460	0.073	1.585 (1.376, 1.829) ***
Healthcare	0.134	0.022	1.144 (1.096, 1.193) ***	0.118	0.022	1.125 (1.077, 1.175) ***	0.114	0.022	1.120 (1.073, 1.170) ***
Technology and resources	0.222	0.037	1.249 (1.161, 1.344) ***	0.196	0.038	1.217 (1.129, 1.312) ***	0.192	0.039	1.212 (1.124, 1.307) ***

Values are presented as odds ratios (OR), 95% confidence intervals (CI). Model 1 was adjusted for age and sex. Model 2 was adjusted for age, sex, education and income. Model 3 was additionally adjusted for age, sex, education, income, smoking, metabolic syndrome. ***p < 0.001.

**Figure 1:**
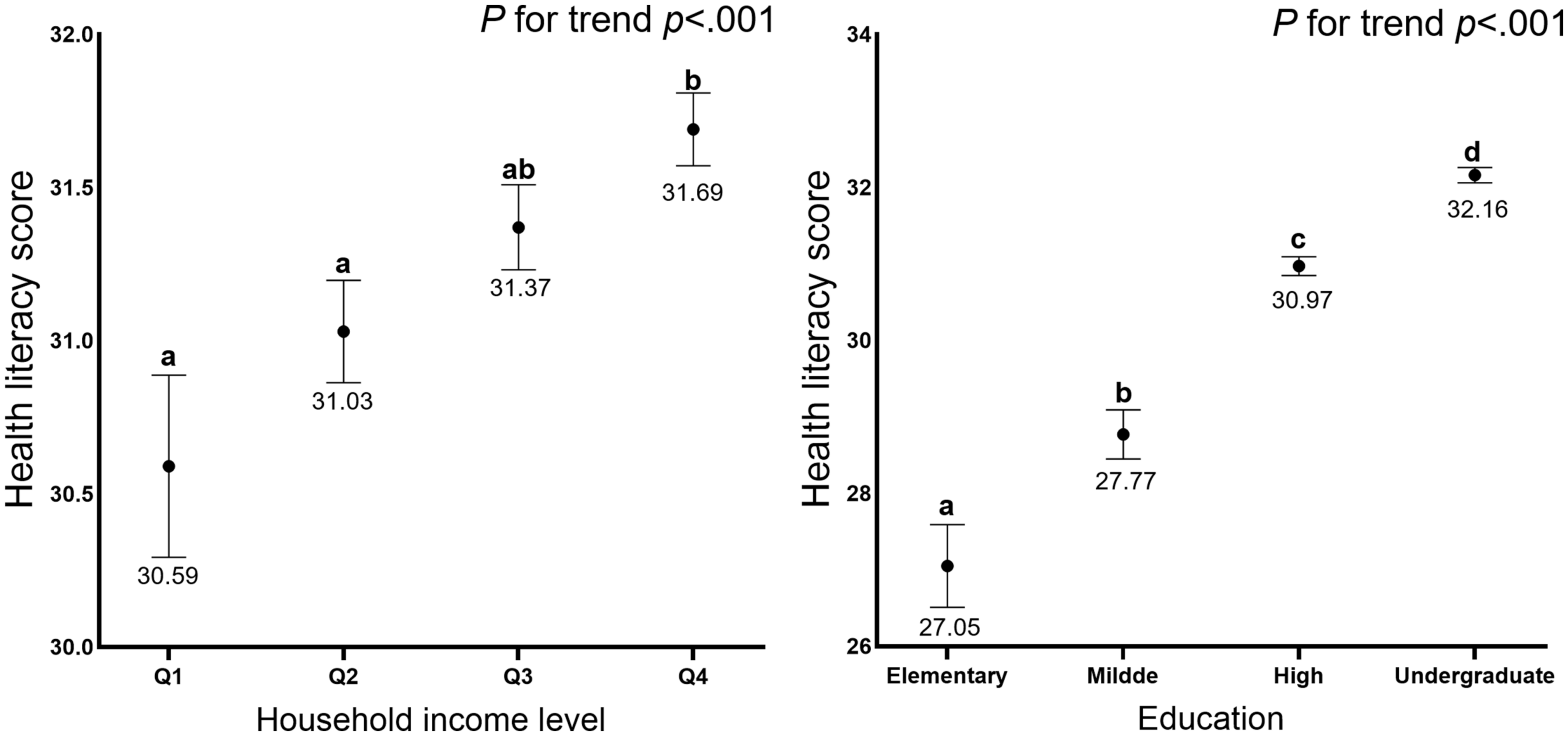
Total health literacy scores across education and household income quartiles. Means with the same letter are not significantly different.

**Figure 2:**
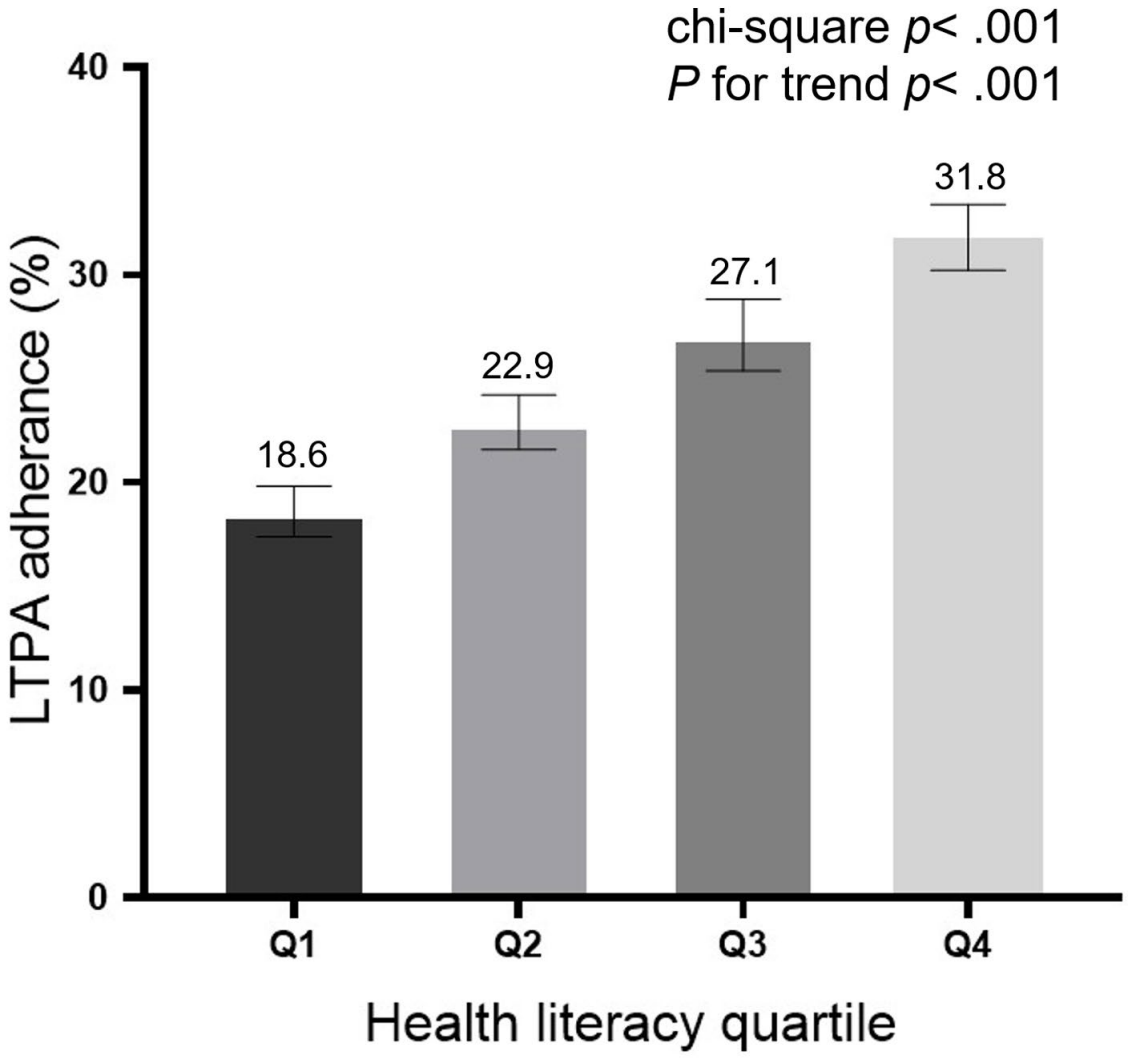
Prevalence of meeting leisure-time physical activity guidelines across health literacy quartiles.

Figure 1 demonstrated socioeconomic gradients in health literacy. Total health literacy scores increased across household income quartiles, from 30.59 in the lowest quartile to 31.69 in the highest quartile (*p* for trend < 0.001). A clear gradient was also observed across education level, with mean health literacy scores increasing from 27.05 in the elementary school group to 27.77 in the middle school group, 30.97 in the high school group, and 32.16 in the undergraduate-or-higher group (*p* for trend < 0.001). Figure 2 further illustrated a graded association between health literacy quartiles and adherence to the LTPA guideline. The prevalence of meeting the guideline increased from 18.6% in Q1 to 22.9% in Q2, 27.1% in Q3, and 31.8% in Q4 (*p* < 0.001). Furthermore, after adjustment for covariates in logistic regression analysis, a significant trend across health literacy quartiles (education level, household income quartile), remained (*p* for trend < 0.001), supporting a positive graded association between health literacy and LTPA adherence.

## Discussion

The present study examined the associations of health literacy with LTPA guideline adherence and socioeconomic inequalities in Korean adults. Three main findings were identified. First, higher total health literacy was independently associated with greater odds of meeting LTPA guidelines. Second, health literacy was socially patterned, with clear gradients across household income and education level, and these gradients were reflected in progressively higher prevalence of LTPA guideline adherence across health literacy quartiles. Third, all four health literacy domains were positively associated with LTPA adherence, with the strongest association observed for the health promotion domain.

These findings extend previous evidence linking health literacy to physical activity. A systematic review reported a generally positive association between health literacy and physical activity, although most available evidence addressed overall physical activity rather than LTPA specifically (9). Although the per-point association of total health literacy with LTPA adherence was modest in magnitude, the cumulative difference across the score range may still be meaningful at the population level. LTPA is a distinct behavioral domain because it is undertaken outside occupational demands and is associated with health outcomes different from those of occupational physical activity (3, 5). Accordingly, our findings suggest that health literacy may be particularly relevant to forms of physical activity that require the ability to interpret health information, apply it in daily life, and sustain behavioral engagement over time. The strongest domain-specific association was observed for the health promotion domain, a finding that is conceptually consistent with the behavioral characteristics of LTPA (19). Individuals with higher health promotion literacy may be better positioned to recognize the benefits of physical activity, interpret health messages, and incorporate activity into daily life. The positive associations observed for the disease prevention, healthcare, and technology and resources domains further indicate that the relationship between health literacy and LTPA is multidimensional rather than confined to a single informational pathway.

Health literacy exhibited socioeconomic gradients, with progressively higher scores across household income quartiles and education levels, particularly across educational attainment. This pattern reinforces the view that health literacy is not merely an individual attribute but a socially patterned resource shaped by unequal access to education, material resources, and opportunities to develop transferable health-related skills (7, 12, 20). The parallel increase in LTPA adherence across health literacy quartiles further suggests that these gradients are behaviorally consequential. The present findings indicate an association between higher health literacy and greater LTPA adherence. Although causality cannot be inferred from the cross-sectional design, the observed pattern suggests that health literacy may be relevant to understanding socioeconomic differences in health-promoting behavior. From a practical perspective, these findings support consideration of health literacy in strategies to promote LTPA. In addition, strategies to increase physical activity have typically emphasized environmental access, risk communication, or motivational counseling. The present findings indicate that health literacy should also be considered a meaningful target for LTPA promotion (21). Interventions that strengthen the ability to access, understand, appraise, and apply health information may help support participation in leisure-time physical activity, particularly among socioeconomically disadvantaged groups (22, 23). The prominent association observed for the health promotion domain further suggests that effective interventions should extend beyond information provision alone and include practical support for interpreting health messages, setting behaviorally actionable goals, and translating knowledge into sustained action.

This study has several notable strengths. It was based on a large, nationally representative sample of Korean adults and examined both total and domain-specific health literacy, enabling a more refined assessment of which dimensions of health literacy were most strongly related to LTPA. Moreover, by demonstrating that socioeconomic gradients in health literacy parallel behavioral differences in LTPA adherence, the study enhances the public health relevance of the findings. Several limitations also warrant consideration. First, because of the cross-sectional design, causality cannot be inferred. Although higher health literacy may promote engagement in LTPA, reverse causality is also possible, whereby sustained participation in health-promoting behaviors contributes to greater health literacy. Second, LTPA, sitting time, and walking time were self-reported and may therefore be affected by recall bias or measurement error. Third, despite adjustment for major covariates, residual confounding remains possible. Factors not captured in the present analysis, such as neighborhood environment, access to recreational spaces, urban or rural residence, psychosocial stress, and social support, may influence both health literacy and LTPA. Fourth, the study population was restricted to adults aged 18 to 64 years, and the findings may not be generalizable to older adults. Fifth, some health literacy domains were assessed using a limited number of items, which may reduce the precision and stability of domain-specific estimates. Finally, the analyses did not account for the complex sampling design of KNHANES, including sampling weights, stratification, and clustering. Therefore, the generalizability of the findings to the national population should be interpreted with caution.

Taken together, the present findings indicate that health literacy is independently associated with LTPA guideline adherence and that this association is socially patterned. Health literacy may represent a modifiable behavioral determinant of LTPA and a potential mechanism linking socioeconomic disadvantage to differences in participation in leisure-time physical activity. Future longitudinal and intervention studies are needed to clarify causality and to determine whether improving health literacy can reduce socioeconomic inequalities in LTPA.

## Data Availability

The datasets presented in this study can be found in online repositories. The names of the repository/repositories and accession number(s) can be found at: KNHANES 2024 data were obtained from the Korea Disease Control and Prevention Agency and are available at: https://knhanes.kdca.go.kr/knhanes.
