# Supplementary material for: Health literacy domains and socioeconomic inequalities in leisure-time physical activity among Korean adults
Source: Front Public Health. 2026 May 28;14:1846028. doi: 10.3389/fpubh.2026.1846028 (PMC13253458; doi:10.3389/fpubh.2026.1846028)
Supplement: Supplementary file 1 [file Table_1.pdf]

Table S1. Detailed descriptions of the health literacy questionnaire items.

Domains	health literacy questionnaire items
Disease prevention	Q1. Can you assess which vaccinations are necessary?
	Q2. Do you understand the degree of risk of mental health issues such as stress and depression?
	Q3. Are you aware of the health symptoms resulting from excessive drinking, smoking and lack of exercise?
Health Promotion	Q4. Can you evaluate how daily behaviors influence health?
Healthcare	Q5. Do you have difficulty understanding explanations and instructions from doctors during medical appointments?
	Q6. Can you assess what needs to be prioritized in case of an emergency?
	Q7. Do you understand how to take medications as explained by doctors or pharmacists?
	Q8. Do you understand patient education materials provided by hospitals?
Technology and resources	Q9. Can you assess the reliability of health information obtained from the internet or media (e.g., television, YouTube)?
	Q10. Can you apply health information obtained from the internet or media (e.g., television, YouTube) to actual health-related behaviors or decision making?
